# Modelling the dynamics of traits involved in fighting-predators–prey system

**DOI:** 10.1007/s00285-015-0869-0

**Published:** 2015-03-14

**Authors:** B. W. Kooi

**Affiliations:** Department of Theoretical Biology, VU University, de Boelelaan 1085, 1081 HV Amsterdam, The Netherlands

**Keywords:** Aggregation technique, Bifurcation analysis, Evolutionary game theory, Hawk–dove game theory, Learning models, Paradox of enrichment, 37G35, 92D15, 92D25, 92D50

## Abstract

We study the dynamics of a predator–prey system where predators fight for captured prey besides searching for and handling (and digestion) of the prey. Fighting for prey is modelled by a continuous time hawk–dove game dynamics where the gain depends on the amount of disputed prey while the costs for fighting is constant per fighting event. The strategy of the predator-population is quantified by a trait being the proportion of the number of predator-individuals playing hawk tactics. The dynamics of the trait is described by two models of adaptation: the replicator dynamics (RD) and the adaptive dynamics (AD). In the RD-approach a variant individual with an adapted trait value changes the population’s strategy, and consequently its trait value, only when its payoff is larger than the population average. In the AD-approach successful replacement of the resident population after invasion of a rare variant population with an adapted trait value is a step in a sequence changing the population’s strategy, and hence its trait value. The main aim is to compare the consequences of the two adaptation models. In an equilibrium predator–prey system this will lead to convergence to a neutral singular strategy, while in the oscillatory system to a continuous singular strategy where in this endpoint the resident population is not invasible by any variant population. In equilibrium (low prey carrying capacity) RD and AD-approach give the same results, however not always in a periodically oscillating system (high prey carrying-capacity) where the trait is density-dependent. For low costs the predator population is monomorphic (only hawks) while for high costs dimorphic (hawks and doves). These results illustrate that intra-specific trait dynamics matters in predator–prey dynamics.

## Introduction

Traditionally in Ecology feeding of the predator population on prey is modelled using the Holling type II functional response which depends on the rate of attack and the time it takes to handle prey. We study the effects of an additional behavioural type via modification of the functional response. The modelling approach has been formulated firstly by Auger and Poggiale (1998). The encouraging example was the study of the dynamics of a population of the domestic cat *Felis catus* with striking differences in many traits between rural and urban cat populations.

This paper deals with the case where part of the captured prey is handled directly and for the other part two predator individuals will fight before consumption. To incorporate this in the model for the trophic interaction between predators and prey, the predator population is divided into three compartments: searcher, feeders and defenders. The ecological processes such as searching and feeding are modelled similar to the classical Holling functional response formulation. In both processes individuals from a single compartment (searchers or feeders) are involved. In a similar way the behavioural processes are modelled, now with encounters between individuals from two different compartments (searchers and feeders) which makes the analysis more complicated. In a number of articles by Auger and Poggiale (1998), Auger et al. (2002, 2006) and recently by Marvá et al. (2013) and Moussaoui et al. (2014), fighting is modelled as a continuous time dynamic hawk–dove game (Maynard-Smith and Price 1973; Maynard-Smith 1982; Hofbauer and Sigmund 1998; Gintis 2000). The gain that individuals receive depends on the amount of prey disputed and the *tactics* that both fighting predator individuals play: hawk or dove. This is encapsulated in a pay-off matrix. The pay-off matrix elements give the outcome of the contest between two individuals playing the same or different tactics. At the population level the *strategy* of the population is quantified by a time dependent *continuous population trait* being the momentary proportion of the population that plays the hawk tactics (frequency dependence). So, we use the term tactics to indicate how predator individuals act during a contest for already captured, but not jet consumed, prey and the term strategy for the performance of a whole population.

In an population dynamics context, evolutionary game theory is often combined with inheritance of traits via mutations at the individual level. Then the time-scale of the dynamical behaviour of the trait is slower than that of the prey and predator populations. Here the trait changes are behavioural, due to for instances imitation or learning, and these processes are often much faster than the rate of change of the population sizes. Nevertheless we will use the same terminology except that we replace mutant by variant.

In the book by Gintis (2000) the *replicator dynamics* (RD) describing the population trait dynamics was derived for a population where the individuals play a hawk–dove game based on a learning process. The reproductive success (number newborns per unit of time) was the fitness criterion while the number of individuals was constant with the fraction hawks as the continuous time trait. An *individual* changes its tactic when its pay-off becomes larger than the average pay-off of the population and in this way the trait of the predator population increases (see for instance in the book by Hofbauer and Sigmund 1998). The solution of the RD formulation gives either a monomorphic predator population consisting of only hawks or a dimorphic predator population where the ratio of the hawks and doves equals the individual gain divided by the costs (see for instance Hofbauer and Sigmund 1998). In both cases the strategy becomes an evolutionary stable strategy (ess).

A reduced predator–prey model was derived by Auger et al. (2006) applying the aggregation technique (Auger et al. 2008) leading to a model with two state variables only: the size of the prey population and the size of the whole predator population instead of three for the sizes of the prey and the hawk predators and dove predators separately. A time-scale separation argument was used where the (searching, feeding and defending) processes at the individual level are faster than the (growth and reproduction) processes at the population level. This resembles the derivation of the “Holling disk-equation”. In this paper we extend these analyses for cases when the time-scale separation is not allowed. Furthermore we re-analyse the periodical oscillation solutions which occur with nutrient enrichment: a phenomenon related to the ’paradox of enrichment’ predicted by the classical predator–prey Rosenzweig–MacArthur models (Rosenzweig and MacArthur 1963).

The main aim of this paper is to compare the RD-modelling described in the books by Hofbauer and Sigmund (1998) and Gintis (2000) with the AD-modelling studied (Metz et al. 1992, 1996; Dieckmann and Law 1996; Geritz et al. 1998, 1999; Dercole et al. 2003; Troost and Kooi 2007), for eco-evolutionary processes here applied for modelling the behavioral (fighting) process. The main reasons to formulate an alternative modelling approach for the RD-modelling are: the definition of fitness is sometimes problematic, a proper mechanistic underpinning for the replicator equation is missing, feedback via the environment is missing, no possibility of evolutionary branching and no mathematical tool to study convergence stability (related to whether an ess is reachable or not). The relationship between these two approaches for discrete time systems with non-overlapping generation was already studied by Dieckmann and Metz (2006). Here we consider, however, a continuous-time system with overlapping generations.

In the AD-approach a variant population of individuals playing an adapted strategy with a trait value close to that of the resident population may invade and successfully replace the resident population leading to a change in the trait. A series of such replacements changes the trait forming a substitution sequence that can lead to convergence to the singular strategy ss: in general a *continuously stable strategy* (css) where the resident population is not invasible by any variant population but here we will also encounter a ss that is a *neutral stable strategy* (nss), see Fig. 9b (Geritz et al. 1998). This process can be formulated as an ordinary differential equation, known as the *canonical equation* of adaptive dynamics (Dieckmann and Law 1996; Dercole and Rinaldi 2008; Kisdi and Geritz 2010). Hence, in the AD-approach, the contest process (fighting between two predator individuals for captured prey individuals) is complementary to the ecological processes (searching, ingestion, assimilation, growth, maintenance, mortality).

We demonstrated previously (Kooi and Troost 2006; Troost and Kooi 2007), that most of the AD problems can be re-formulated in bifurcation theory quite naturally, which increases generality and allows the inclusion of ecological implications [(for instance (co-)existence, oscillatory dynamics]. The trait values of the resident and the variant population are the bifurcation parameters. The so called *pip-plot*s (pairwise invasibility plot Geritz et al. 1998) show in the trait space, spanned by the resident and variant traits, where either the resident population wins, the variant population wins or both populations coexist. These regions are separated by transcritical bifurcation curves. Moreover we also include Hopf bifurcation curves in the pip-plots. Then in some regions of the trait space the resident or the variant population wins or both populations coexist while the predator–prey system oscillates periodically.

The long-term dynamics of the predator–prey system is obtained by numerical bifurcation analysis. Similar as in the predator–prey Rosenzweig-MacArthur model (Rosenzweig and MacArthur 1963) for low carrying capacities there is a stable equilibrium for the prey population alone. When the prey carrying capacity is sufficiently high, a stable coexistence of both prey and predator populations either at equilibrium or a limit cycle when the carrying capacity is very high.

We will here demonstrate that he RD- and the AD-approach lead to the same results for the system in equilibrium whereby in the RD-approach there is an ess and in the AD-approach a nss. When periodical predator–prey solutions occur for higher carrying capacities, the AD-approach results differ from those for the RD-approach. In the AD-approach there is a css for a monomorphic prey population while in the RD-approach during one period switches occur between monomorphism and dimorphism back and forth when the amplitude of the limit cycle is sufficiently large. The introduction of an addition stage in feeding prey (namely fighting besides feeding) appears to have a stabilizing effect that is depending of the costs. Furthermore, elaboration of the AD-approach yields that two predator populations with different trait values and feeding on a single prey population can coexist stably: contrary to the competitive exclusion principle.

## Problem formulation and analysis methods

We consider a predator population p, with density $p(t)$ feeding on a prey population n, with density $n(t)$ model. The prey grows logistically in absence of the predator. A searching predator S individual becomes feeder F after meeting a prey individual (second order mass action process with rate $a$). It starts to handle the prey but does not directly digests it.

If during this handling phase the individual does not encounter a searching predator it digests the prey (including growth and/or reproduction) and becomes a searcher again (first order process with rate $\beta$). These Holling type II searching and feeding processes are described using a pseudo-reaction scheme as follows:

$$\begin{aligned} \text {S} +\text {n}&\mathop {\longrightarrow }\limits ^{a} \text {F},\quad \text {F} \mathop {\longrightarrow }\limits ^{\beta } \text {S} \end{aligned}$$

Observe that the encounters are inter-specific between predator individuals and prey individuals.

On the other hand if during this handling phase the individual encounters a searching predator (second order mass action process with rate $b$) they become defenders D. After fighting and handling they digest their gain (including growth and/or reproduction) and become searcher S again (first order process with rate $\gamma$). The gain depends on whether the individual plays the hawk H or dove D tactics and therefore we distinguish six compartments, searcher, feeders and defenders each either hawk or dove: S $=$  SH $+$ SD, F $=$ FH $+$ FD and D $=$ DH $+$ DD where the first letter indicates the stage S, F or D and the second the tactics (H or D).

Then searching and feeding processes are described using

$$\begin{aligned} \text {SH} +\text {n}&\mathop {\longrightarrow }\limits ^{a} \text {FH},\quad \text {FH} \mathop {\longrightarrow }\limits ^{\beta } \text {SH}\\ \text {SD} +\text {n}&\mathop {\longrightarrow }\limits ^{a} \text {FD},\quad \text {FD} \mathop {\longrightarrow }\limits ^{\beta } \text {SD} \end{aligned}$$

and fighting for captured prey is described using a pseudo-reaction scheme as follows:

$$\begin{aligned} \text {FH} +\text {S}&\mathop {\longrightarrow }\limits ^{b} \text {DH}\\ \text {SH} +\text {F}&\mathop {\longrightarrow }\limits ^{b} \text {DH},\quad \text {DH} \mathop {\longrightarrow }\limits ^{\gamma } \text {SH}\\ \text {FD} +\text {S}&\mathop {\longrightarrow }\limits ^{b} \text {DD}\\ \text {SD} +\text {F}&\mathop {\longrightarrow }\limits ^{b} \text {DD},\quad \text {DD} \mathop {\longrightarrow }\limits ^{\gamma } \text {SD} \end{aligned}$$

Observe that these encounters are intra-specific and that the process rates for the different combinations are the same.

The profit for the two fighting individuals after the contest is given in the so called pay-off matrix introduced in game theory (Hofbauer and Sigmund 1998)

1 $$\begin{aligned}&\begin{matrix} \qquad DH \qquad DD\end{matrix}\nonumber \\ \mathbf {A}=&{\begin{pmatrix} (G-C)/2 &{}\quad G \\ 0 &{}\quad G/2 \end{pmatrix}}\quad \begin{matrix}DH \\ DD, \end{matrix} \end{aligned}$$

where $G$ is the gain and $C$ the costs. A continuous-time version of this classical hawk–dove game is used (see also Gintis 2000). The gain (now per time) for the individuals from this contests is obtained using the elements of the pay-off matrix depending on the *tactics* of the individual and that of the opponent. The hawk–dove *strategy* of a population consisting of individuals playing the hawk or dove tactics, is described by the proportion of hawks, a scalar, being the *continuous trait* of this *frequency-dependent* problem.

We will formulate and discuss two models to describe the dynamics of this (in our case predator) population trait: the RD- and the AD-approach. Crucial is the definition of the fitness and the trait besides the functional dependency of the fitness on the trait (see also McGill and Brown 2007). In general the fitness of the population $W$ is formulated as a function of the trait value of a variant (individual or population) $\rho ^m$, the trait value of the resident population $\rho ^r$ and the size of this population $p$ (which in our case depends also on prey density $n$, but in general also on other environmental factors): that is $W(\rho ^m,\rho ^r,p)$.

In life history theory evolution the fitness $W(\rho ^r)$ is maximized. The trait is a life history characteristic, for instance investments in reproduction, and a suitable fitness function is for instance the per capita growth rate. Then

2 $$\begin{aligned} \frac{dW}{d \rho ^r}=0, \end{aligned}$$

is solved using optimization methods.

In the evolutionary game theory the fitness, $W(\rho ^m,\rho ^r)$, is the rate of invasion of a variant individual into the resident population. It depends on the trait of a variant individual $\rho ^m$ and the trait of the resident population $\rho ^r$ which is the average trait value of the resident population individuals when the resident population shows variation. The size of the population $p$ is constant and no feedback from the environment is take into account. That is the behavioural learning process occurs as if the defending population is isolated from the rest of the predator–prey system.

The temporal change in the trait is in the direction of and proportional to the slope of the fitness function, $\partial W/\partial \rho ^m$. An ess is the trait value where the fitness is highest and where no variant individual can invade. So, the variant individual is not interacting in a pair-wise fashion with other individuals but interacts with the whole population having a average trait. The fitness measures the rate of invasion of a rare variant individual into the resident population. Then the ess where $\rho ^r=\rho ^*$ is obtained at:

3 $$\begin{aligned} \frac{\partial W(\rho ^m,\rho ^r) }{\partial \rho ^m}\big |_{\rho ^r=\rho ^*}=0 \end{aligned}$$

evaluated at $\rho ^*$ while

4 $$\begin{aligned} \frac{\partial ^2 W(\rho ^m,\rho ^r) }{\partial \rho ^{m2}}\big |_{\rho ^r=\rho ^*}<0 \end{aligned}$$

guarantees an evolutionary stable equilibrium ess. Observe that resistance to invasion here says nothing about what would happen if the population starts nearby this point. Often this ess point is also the equilibrium of the *replicator equation* (see Hofbauer and Sigmund 1998) which is a ordinary differential equation for the trait $\rho ^r$:

5 $$\begin{aligned} \frac{d\rho ^r}{dt}&=\rho ^r \bigl (W(\rho ^m,\rho ^r)-\overline{W}(\rho ^r)\bigr ), \end{aligned}$$

where $\overline{W}(\rho ^r)$ is the average fitness of the population or, when the resident population shows variation, the fitness of an individual with the average trait $\rho ^r$.

In the RD-approach the ecological model and the contest model are kept separated because the size of the population is kept constant with the derivation of the replicator equation. When the trait dynamics can be assumed to be fast we have an algebraic expression for the trait as a function of the gain and costs (probably time-dependent). That is, the ess trait value is then adapted instantaneously to the gain changes (costs are constant here).

In the adaptive dynamics (AD) theory the fitness, $W(\rho ^m,\rho ^r,p)$, is now the rate of invasion, generally denoted by $s$, of a rare variant population into the resident population. Now the population is not strictly constant in size and all interactions between the populations and the environment are taken into account. The canonical equation derived by Dieckmann and Law (1996) describes the dynamics of the resident trait as a continuous flow in the trait space by

6 $$\begin{aligned} \frac{d\rho ^r}{dt}=k\frac{\partial W(\rho ^m,\rho ^r,p)}{\partial \rho ^m}\big |\rho ^r. \end{aligned}$$

This formulation is derived as a deterministic approximation of a sequence of trait substitutions as a random walk in the trait space determined by the processes of mutation and selection (competition). The rate constant $k$ gives the “speed” of the time-evolution where trails are random (see Dieckmann and Law 1996). This factor describes characteristics of the random mutational process of choosing the variant trait value $\rho ^m$ for the next evolutionary step. In conclusion, the trait changes with a rate proportional to the fitness gradient and the endpoints is reached when the fitness gradient is zero.

Hence, a ss trait value is fixed by a zero fitness gradient and the same expression as Eq. (3) applies. By Geritz et al. (1998) eight different ss’s are distinguished based on the second derivatives at the point which differ in general from Eq. (4). It appears that the slope of the non-trivial zero contour line evaluated at a ss determines for the monomorphic population already the evolutionary dynamics in the neighbourhood of the ss. These eight different ss’s (Geritz et al. 1998) were illustrated using pip-plots. These plots show in the trait space $(\rho ^m,\rho ^r)$ where either the resident population wins, the variant population wins or both populations coexist all in equilibrium or periodic solution of the full ecological system. These regions are separated by zero growth curves.

According to Feldman and Aoki (2006), Aoki and Feldman (2006) learning is a means of acquiring information about the environment and of expressing a phenotype (behavior) appropriate to that environment. Two forms of learning are distinguished by the source of the information acquired. Individual learning (IL) occurs when an organism depends on its personal experience to gather the information directly from the environment. The second form of learning is social learning (SL), which occurs when an organism obtains the information indirectly by copying other organisms, e.g., by imitation. With learning, choosing the variant trait values can be either randomly (like mutations) or deliberately based on the assessment of the outcome. In the book by Hofbauer and Sigmund (1998) it is mentioned that when traits are myopic in learning models the variant trait values are only in the immediate vicinity of the resident trait value. When furthermore allowing infinitesimal small continuous trait changes this could be implemented by reducing $k$ when the ss-point is approached. Note that generally in the AD formulation only small but discrete mutational steps are assumed (Geritz et al. 1998).

Although we use terminology from evolutionary game theory we apply the same ideas here for the analysis of ecological behaviour processes. With behavioral processes such as fighting, the strategic change of the trait is by learning, using the outcome of the contests. These changes occur at a time scale relatively fast with respect to the ecological population time scale, in contrast to genetic changes by mutations at the generation time scale. Notwithstanding that the biological motivation and justification of the applicability of the approaches differ, the mathematical and analysis tools are the same.

To analyse these models we use bifurcation theory which focuses on the qualitative changes in the stability and type of asymptotic solutions of the system (steady states, periodic cycles, chaos) under parameter variation. The basics of bifurcation analysis are described in the books (Guckenheimer and Holmes 1985; Wiggins 1990; Kuznetsov 2004). Examples of ecological applications of bifurcation analysis are given in (Bazykin 1998; Kooi 2003) and of AD in (Dercole and Rinaldi 2008; Kooi and Troost 2006; Troost and Kooi 2007). The asymptotic behaviour of the model has been analyzed using the symbolic analysis software Maple (Maple 2008) and the numerical bifurcation analysis software auto (Doedel and Oldeman 2009). With the studied predator–prey model we found only the transcritical bifurcation (for equilibria and limit cycles), $TC$, and the Hopf bifurcation, $H$. At a $TC$ point one population invades an existing system while at a $H$ bifurcation an equilibrium of the system looses its stability and a limit cycle emerges under parameter variation.

In the RD-approach the governing equations are all ode’s whether the replicator equation, which is an ode to describe the dynamics of the trait, is used or directly the quasi-static ess trait value is substituted in the predator–prey system. The same type of bifurcation analysis can be done as by Auger et al. (2006). For the AD-approach application of the bifurcation theory is different when the time-scale separation argument holds or not. When not and the canonical equation is used then again all the equations are ode’s, see Dercole and Rinaldi (2008) for many examples. When the learning process is fast, however, the outcome of the competition between the resident and variant, treated as long-term dynamics results, are presented in pip-plots where the two traits of the resident and variant are the bifurcation parameters. The ss point is the intersection of two transcritical bifurcation curves. Then, one can still do one bifurcation analysis for the behavioural and ecological model combined. This technique was already explored by Kooi and Troost (2006) and Troost and Kooi (2007) and will be elaborated below.

## Mathematical model formulation and results

In this section we formulate the models and analyse the resulting equations. The RD-approach and the AD-approach will be discussed in sequence.

### Replicator dynamics (RD) model

**Fig. 1:**
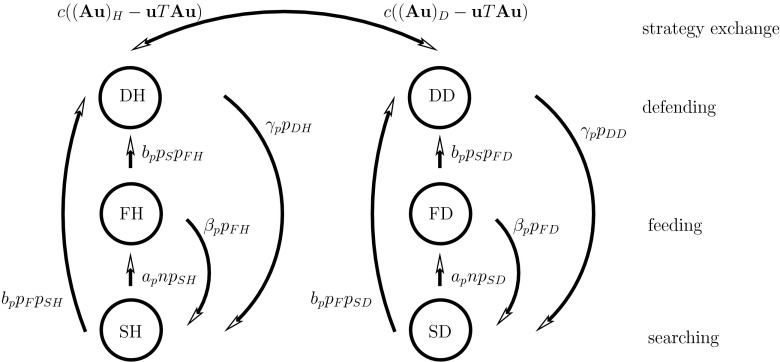
The predator fluxes between the six compartments in the RD-approach. The fluxes except those between defending individuals, are due to encounters between predator individuals in a different behavior stage or between a predator individual and a prey individual modeled by the law of mass-action. The fluxes between the two defending stages are due to the change of tactics (hawk or dove)

Figure 1 illustrates the fluxes between the six predator compartments: SH, SD, FH, FD, DH and DD. The fluxes between the sub-populations follow from mass-action arguments or game theoretical expressions for the outcome of the contests in the defending stage where the individuals can change their tactics via learning and subsequently change the strategy of the predator population.

The pay-off matrix for the contest between two individuals in the defender state are that of the hawk–dove model given in Eq. (1). The gain $G$ is the average amount of prey individuals that two predators dispute per unit of time. When two hawks fight, they can get wounded. Let $C$ be the cost due to fighting between hawks per a pair of defending predators and per unit of time. $C$ is a positive parameter which is allowed to be larger than the gain $G$. The strategy of a population is determined by the proportion of hawks in the population treated as its trait $\rho$.

The outcome of these individual contests leads to the following game theoretical model for the population of defenders $p_D=p_{DH}+p_{DD}$ with proportions hawk $\rho =p_{DH}/p_D$ and doves $1-\rho =p_{DD}/p_D$ with pay-off matrix $\mathbf {A}$ Eq. (1) and vector $\mathbf {u}= (\rho ,1-\rho )^T$:

7a $$\begin{aligned} (\mathbf {A}\mathbf {u})_H&= \frac{G-C}{2} \frac{p_{DH}}{p_D} + G \frac{p_{DD}}{p_D}=\rho \frac{G-C}{2}+ (1-\rho ) G,\end{aligned}$$

7b $$\begin{aligned} (\mathbf {A}\mathbf {u})_D&= \frac{G}{2} \frac{p_{DD}}{p_D}=(1-\rho ) \frac{G}{2}, \end{aligned}$$

and the average gain for the whole population reads

8 $$\begin{aligned} \mathbf {u}^T\mathbf {A}\mathbf {u}&=\frac{p_{DH}}{p_D} (\mathbf {A}\mathbf {u})_H+ \frac{p_{DD}}{p_D}(\mathbf {A}\mathbf {u})_D =\frac{G}{2}- \frac{C}{2}\frac{p_{DH}^2}{p_D^2}=\frac{G}{2}-\rho ^2 \frac{C}{2}. \end{aligned}$$

From Eqs. (7, 8) we derive

9 $$\begin{aligned} \bigl ((\mathbf {A}\mathbf {u})_D - \mathbf {u}^T\mathbf {A}\mathbf {u}\bigr )p_{DD} + \bigl ( (\mathbf {A}\mathbf {u})_H - \mathbf {u}^T\mathbf {A}\mathbf {u}\bigr )p_{DH}=0. \end{aligned}$$

The equations for the defending population $p_D$ related to changing the strategy read (Auger et al. 2006)

10a $$\begin{aligned} \frac{dp_{DD}}{d\tau }&=c\bigl ( (\mathbf {A}\mathbf {u})_D - \mathbf {u}^T\mathbf {A}\mathbf {u}\bigr ) p_{DD}=-\frac{c}{2}\rho (G-\rho C)p_{DD},\end{aligned}$$

10b $$\begin{aligned} \frac{dp_{DH}}{d\tau }&=c\bigl ( (\mathbf {A}\mathbf {u})_H - \mathbf {u}^T\mathbf {A}\mathbf {u}\bigr ) p_{DH}=\frac{c}{2} (1-\rho )(G-\rho C)p_{DH}, \end{aligned}$$

where $c$ is a rate-coefficient of the change of tactics. We recall that $(\mathbf {A}\mathbf {u})_D$ (resp. $(\mathbf {A}\mathbf {u})_H$) represents the average prey density that dove (resp. hawk) predators dispute per unit of time. We must multiply these quantities by their densities to obtain the total prey density density ingested by the dove and hawk searching sub-populations yielding their gain per unit of time. Hence both populations sizes $p_{DH}, p_{DD}$ become time independent when their average gain Eq. (7) equals the average of the whole population Eq. (8). Adding the two equations Eq. (10) gives, using Eq. (9), that the densities of defending predators individuals $p_D=p_{DH}+p_{DD}$ does not change due to the fact that some individuals alter their tactics. Then we get the classical *replicator dynamics equation* for the continuous trait $\rho$, the proportion of density of hawks:

11 $$\begin{aligned} \frac{d\rho }{d\tau }&=c\bigl ( (\mathbf {A}\mathbf {u})_H - \mathbf {u}^T\mathbf {A}\mathbf {u}\bigr ) \rho =\frac{c}{2}\rho (1-\rho )(G-\rho C) . \end{aligned}$$

This replicator dynamics equation has three equilibria $\rho ^*=0$, $\rho ^*=1$, $\rho ^*=G/C$. Since $\rho ^r\in [0,1]$ and we assume $C>0$ and $G\ge 0$ this yields the ess:

12 $$\begin{aligned} \rho ^*=\frac{p_{DH}}{p_{D}}=\left\{ \begin{array}{ll} \frac{G}{C} &{}\quad \text {if}\, 0\le G<C \\ 1 &{}\quad \text {if}\, G>C. \end{array}\right. \end{aligned}$$

By Auger et al. (2006) the following model is formulated and analysed:

13a $$\begin{aligned} \frac{dn}{d\tau }&= \varepsilon \bigl (r n (1-\frac{n}{K})- anp_S \bigr ),\end{aligned}$$

13b $$\begin{aligned} \frac{dp_{SD}}{d\tau }&= -b p_F p_{SD} - a n p_{SD} + \beta p_{FD} + \gamma p_{DD} \nonumber \\&\quad +\varepsilon \bigl (\alpha \,( \beta p_{FD}+ ( \mathbf {A}\mathbf {u})_Dp_{DD})- \mu p_{SD}\bigr ), \end{aligned}$$

13c $$\begin{aligned} \frac{dp_{FD}}{d\tau }&= -b p_S p_{FD} + a n p_{SD}- \beta p_{FD} -\varepsilon \mu p_{FD},\end{aligned}$$

13d $$\begin{aligned} \frac{dp_{DD}}{d\tau }&= b p_F p_{SD} - \gamma p_{DD}+b p_S p_{FD}+ c p_{DD} \bigl ( (\mathbf {A}\mathbf {u})_D - \mathbf {u}^T\mathbf {A}\mathbf {u}\bigr ) -\varepsilon \mu p_{DD},\end{aligned}$$

13e $$\begin{aligned} \frac{dp_{SH}}{d\tau }&= -b p_F p_{SH} - a n p_{SH}+\beta p_{FH} +\gamma p_{DH} \nonumber \\&\quad + \varepsilon \bigl (\alpha \,( \beta p_{FH}+ (\mathbf {A}\mathbf {u})_Hp_{DH})-\mu p_{SH}\bigr ),\end{aligned}$$

13f $$\begin{aligned} \frac{dp_{FH}}{d\tau }&= -b p_S p_{FH} + a n p_{SH} -\beta p_{FH} -\varepsilon \mu p_{FH},\end{aligned}$$

13g $$\begin{aligned} \frac{dp_{DH}}{d\tau }&= b p_F p_{SH} - \gamma p_{DH} + b p_S p_{FH} + c p_{DH} \bigl ( (\mathbf {A}\mathbf {u})_H - \mathbf {u}^T\mathbf {A }\mathbf {u}\bigr ) - \varepsilon \mu p_{DH}. \end{aligned}$$

where $\alpha$ is the prey–predator efficiency coefficient and $\mu$ the predator death rate. We assume two different time scales. The fast time scale (with fast time variable $t$) corresponds to the inter-specific searching and handling for the prey by the predators. The slow time scale (with slow time variable $\tau$) corresponds to the (logistic) growth of the prey population and mortality of the predator. The parameter $\varepsilon$ controls the degree of time separation.

The first contribution in Eqs. (13d) and (13g) is the flux of prey density when dove (resp. hawk) predators return from finding to searching without encountering another individual. These prey density flux are $\beta p_{FD}$ for doves (resp. $\beta p_{FH}$ for hawks). The second contribution in Eqs. (13b) and (13e) corresponds to prey density obtained as gain by fighting: $(\mathbf {A}\mathbf {u})_Dp_{DD}$ for doves (resp. $(\mathbf {A}\mathbf {u})_Hp_{DH}$ for hawks) and given in Eq. (7). The contribution described by Eqs. (13d) and (13g) correspond to the strategy exchange which is given in Eq. (10).

Summation of the searchers $p_S$, feeders $p_F$ and defenders $p_D$ in Eqs. (13b–13g) gives

14a $$\begin{aligned} \frac{dn}{d\tau }&= \varepsilon \bigl (r n (1-\frac{n}{K})- anp_S \bigr ),\end{aligned}$$

14b $$\begin{aligned} \frac{dp_{S}}{d\tau }&= -b p_F p_{S} - a n p_{S} + \beta p_{F} + \gamma p_{D} +\varepsilon \bigl (\alpha \,( \beta p_{F}+ ( \mathbf {u}^{T}\mathbf {A}\mathbf {u})p_{D})- \mu p_{S}\bigr ), \end{aligned}$$

14c $$\begin{aligned} \frac{dp_{F}}{d\tau }&= -b p_S p_{F} + a n p_{S}- \beta p_{F} -\varepsilon \mu p_{F},\end{aligned}$$

14d $$\begin{aligned} \frac{dp_{D}}{d\tau }&= 2 b p_F p_{S} - \gamma p_{D}- \varepsilon \mu p_{D} , \end{aligned}$$

together with Eq. (8) that describes the term $\mathbf {u}^{T}\mathbf {A}\mathbf {u}$. Hence, reduction to the four dimensional system for $n$, $p_S$, $p_F$ and $p_D$ is possible. By Auger et al. (2006) the following expression for the gain $G$ was derived being the amount of prey a *single* defending predator disputes, which is the amount of prey per unit of time that is obtained by a *pair* of defending predators

14e $$\begin{aligned} G=2 b \frac{p_F p_{S}}{p_{D}} . \end{aligned}$$

Observe that in this paper the gain $G$ is defined as in Auger et al. (2006), Eq. (8) and not as in Auger et al. (2002), Eq. (2). This gain (in general density-dependent) is used to calculate the average gain for the whole population:

14f $$\begin{aligned} \mathbf {u}^{T}\mathbf {A}\mathbf {u}&=\left\{ \begin{array}{ll} \frac{G}{2}-\rho ^{2} \frac{C}{2} &{}\quad \text {if}\, G<C \\ \frac{G}{2}- \frac{C}{2} &{}\quad \text {if}\, G>C, \end{array} \right. \end{aligned}$$

where the dynamics of the trait $\rho$ is described by the replicator equation (an ode) given in Eq. (11). Observe that using Eq. (14d) we get in equilibrium

15 $$\begin{aligned} G^*=\gamma +\varepsilon \mu , \end{aligned}$$

and this expression will be used below with the analysis of equilibrium states.

When the time-scale separation argument is used, the processes described in Eqs. (14) by the expressions on the right-hand sides and before the $\varepsilon$ terms, run at the fast time scale and model, at the individuals level, the dynamics in the searching, feeding and defending stages. The terms multiplied by a factor $\varepsilon$ run at the slow time scale and model the population dynamics with growth and/or reproduction, and death. Hence for $\varepsilon =0$ the fast time scale formulation applies yielding the reduced model formulation discussed by Auger et al. (2006) and in the Appendix, while $\varepsilon =1$ (and $\tau =t$) in the full model formulation.

### RD-results

The long-term dynamics predicted by the RD-models are presented in two parameter bifurcation diagrams where the game costs $C$ and the prey’s carrying capacity $K$ as free parameters.

**Fig. 2:**
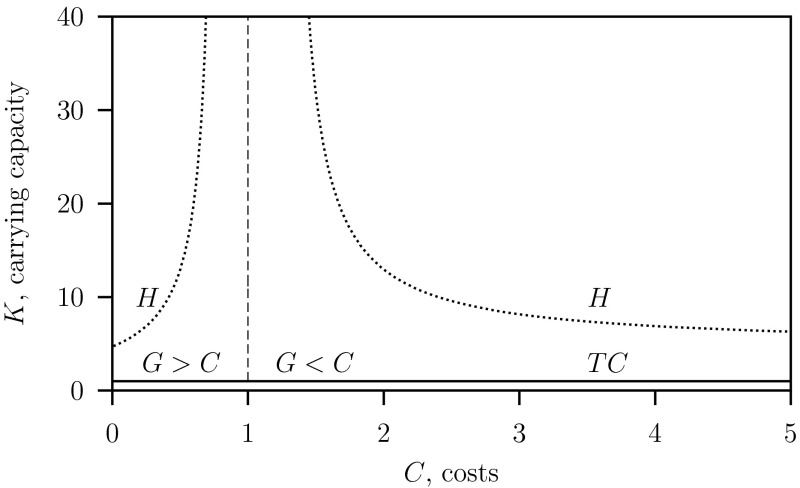
Two-parameter bifurcation diagram $C$ versus $K$ for the reduced system Eq. (28) where $\varepsilon = 0$ with $G$ given in Eq. (27c). Below the transcritical bifurcation curve $TC$ only the prey survives and above this curve there is bith prey and predator exist. In the region between the $TC$ curve and the Hopf bifurcation curve $H$ a stable equilibrium and above the curve $H$ a stable limit cycle exist. The dashed vertical curve at $C=1$ separates the regions were the predator population is monomorphic $G>C$ and dimorphic $G<C$. Parameter values as given in Table 1

In Fig. 2 the results are shown in for the reduced predator–prey system Eq. (28) where $c=\infty$ and $\varepsilon =0$. This model is described by Auger et al. (2006) and in the Appendix. The parameter values are given in Table 1. The rate of change of the tactics is fast, $c=\infty$, so that we can use for the gain equilibrium Eq. (27c), that is $G=\gamma =1$. These results were already obtained and discussed by Auger et al. (2006) and are used here for comparison.

**Table 1:** List of symbols for variables and parameters used in the text

Symbols	Value	Description
$n,p$	Variable	Population: prey, predators
$p_{i},p_{j},p_{ij}$		Predator stage sizes, $i\in \{D,H\}$ and stage $j\in \{S,F,D\}$
$S,F,D$	–	Stage: searching, feeding, defending
$D,H$	–	Tactics: dove, hawk
$r,m$	–	Population: resident, variant
$a$	1	Encounter rate between searcher individuals and prey
$b$	1	Encounter rate between predator individuals
$c$	$\infty$	Rate of change of tactics (RD) or trait (AD)
$C$	Variable	Costs rate in game dynamics
$G$	1	Gain rate in game dynamics
$K$	Variable	Prey carrying capacity
$r$	1	Prey intrinsic growth rate
$s_e,s_c$	Variable	Invasion rate for equilibrium and limit cycle
$T_0$	Variable	Period of a limit cycle
$t$	Variable	Slow time
$\alpha$	1	Efficiency coefficient conversion prey–predator
$\beta$	1	Predator feeding rate (reciprocal of handling time)
$\gamma$	1	Intra-specific predator fighting rate
$\delta$	0.02	Numerical parameter
$\varepsilon$	$0$ or $1$	Rate of inter-specific predator–prey interaction
$\rho$	Variable	Trait
$\mu$	0.5	Predator death rate
$\tau$	Variable	Fast time

Stages: $S,F,D$ and defender individual tactics: $D,H$. The strategy of the populations are described by the trait $\rho$ the proportion of defender individuals playing the hawk tactic. Notice that in this theoretical study the parameter values are not related to a specific application and therefore the units are not given

**Fig. 3:**
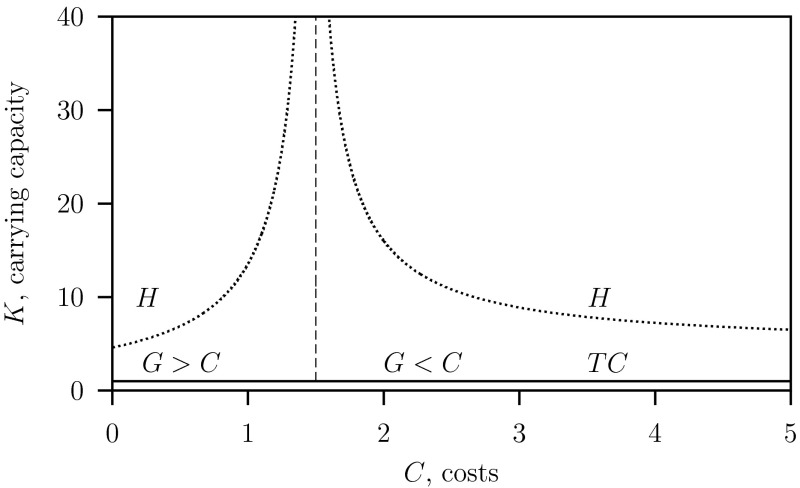
Two-parameter bifurcation diagram $C$ vs $K$ for the system Eq. (14) where $\varepsilon = 1$ with $G$ given in Eq. (14e). Parameter values as given in Table 1 and see Fig. 2 for an explanation of the symbols

The bifurcation diagram Fig. 3 is obtained for the system Eq. (14) where $\varepsilon =1$ and $c=\infty$, that is with a quasi-static ess trait $\rho ^*$ given by Eq. (12). Observe that the bifurcation pattern is the same as that of the reduced system Eq. (28) shown in Fig. 2. Here the size of the gain changes when the costs are varied and therefore the gain is now given by Eq. (14e) instead of Eq. (27c). Consequently the separator where the population switches from monomorphic ($G>C$) to dimorphic ($G<C$) or visa verse, occurs at $C=G=\gamma +\varepsilon \mu$ (see Eq. (15)) and not as in Fig. 2 where it occurs at $C=G=\gamma$ (see Eq. (27c)). Here for $K=2$ and all other parameter given in Table 1, at $C=\gamma +\varepsilon \mu =1.5$ instead of $C=\gamma =1$ as in (Auger et al. 2006). The Hopf bifurcation curves are at higher carrying capacity $K$ values, so there is a stable equilibrium in a larger $C$–$K$ parameter region.

The solutions of the system Eq. (14) where $\varepsilon =1$ and $c=\infty$ where close to those for the system with $c=1$ (results are not shown). Furthermore only slightly different results occur when the system is periodically oscillating. Hence, the assumption using a quasi-steady state trait value Eq. (12) where learning is fast instead of the trait described by a replicator equation Eq. (11) is justified.

**Fig. 4:**
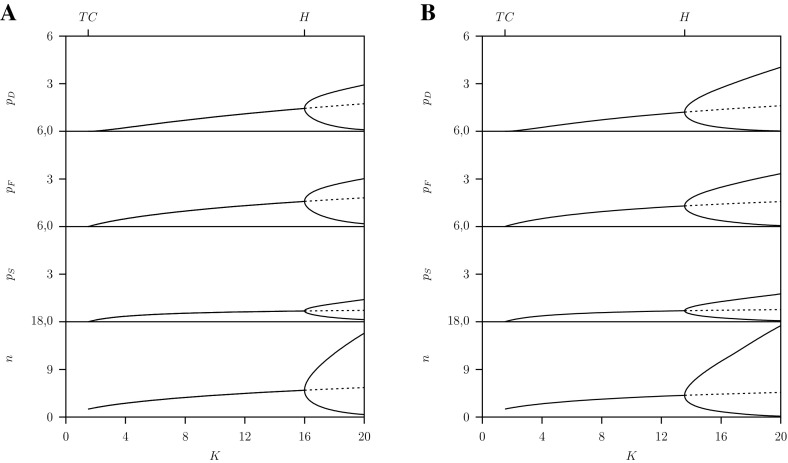
One-parameter diagrams for the prey population $n$ and predator sub-population densities $p_S$, $p_F$, $p_D$ as function of the carrying capacity $K$ for the RD-approach, system Eq. (14). Below the transcritical bifurcation point $TC$ all predator sub-populations are zero: that is the predator population is extinct. Between $TC$ and the Hopf bifurcation point $H$ a stable equilibrium and above $H$ a stable limit cycle exists. Parameter values as given in Table 1 where A: $C=2$ and B: $C=1$

We finish the discussion of the RD system with showing the one-parameter diagram with the prey’s carrying capacity $K$ as free parameter for the densities $n, p_S, p_F, p_D$ where $\varepsilon =1$, $c=\infty$ and $C=2$ the same as shown in Fig. 4a. Here we started from the stable equilibrium at $K=2$. For decreasing $K$ at the transcritical bifurcation $TC$ the predator population goes extinct whereby all three sub-populations disappear simultaneously. For values above the Hopf bifurcation $H$ where the equilibrium is unstable and stable limit cycles occur. In this dimorphic case where $G<C$ the trait is time dependent and is adapted instantaneously. For $C=1$ where we are in the region where $G>C$ and the population is monomorphic, the dynamical patterns are shown in Fig. 4b. However, with increasing $K$ the amplitude of the limit cycle solution increases and at about $K=16.63$ during one period switches occur between monomorphism and dimorphism back and forth.

We will use the results given in Figs. 3 and 4 for comparison of the results of the RD versus AD approach in Sect. 4.

### Adaptive dynamics (AD) model

**Fig. 5:**
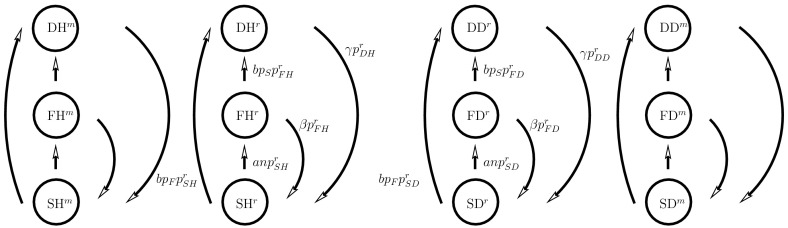
The predator fluxes between the twelve compartments in the AD approach where each of the six resident compartments have a variant version. Compare this scheme with that for the RD approach given in Fig. 1. Here, a searching resident individual in $p_{SH}^r+p_{SD}^r$ can encounter a feeding variant individual in $p_{FH}^m+p_{FD}^m$ and both move all to their defending states $p_{DH}^r$, $p_{DD}^r$, $p_{DH}^m$ and $p_{DD}^m$ respectively, and start to fight. The same holds for a searching variant individual which encounters a feeding resident individual

Figure 5 illustrates the fluxes between the compartments where we augmented the resident system, shown in Fig. 1, in order to account for the variant population dynamics. The single difference between the resident and the variant population is their trait value denoted by $\rho ^r$ and $\rho ^m$, being the proportions of the hawk individuals in the defending stage. For the resident sub-population the $p$-fractions are indicated by a superscript $r$ and for the variant sub-populations by a superscript $m$. The fluxes between the sub-populations include interactions between the searching and feeding variant and resident individuals by introducing $p_S=p_{SD}^r+p_{SH}^r+p_{SD}^m+p_{SH}^m$ and $p_F=p_{FD}^r+p_{FH}^r+p_{FD}^m+p_{FH}^m$.

There is in the AD approach no change of tactics in the defending stage as was the case in the RD approach. In the AD-approach this change is due to competition between the resident and a variant population when the variant population invades and replaces the resident population. Hence, in order to find the solution for the game equilibrium we test whether the variant being rare can invade the system consisting of the prey and the resident predator. Similar to the fast rate of change of tactics in the RD approach where $c=\infty$, here we will assume that the trait value changes due to the competitions outcome occur at a fast time scale.

The equations for the predator–prey system are:

16a $$\begin{aligned} \frac{dn}{d\tau }&=\varepsilon \bigl ( rn(1-\frac{n}{K}) - an(p_{SD}^r+p_{SH}^r)- an( p_{SD}^m+p_{SH}^m)\bigr ), \end{aligned}$$

16b $$\begin{aligned} \frac{dp_{SD}^j}{d\tau }&= -b p_F p_{SD}^j-a n p_{SD}^j+\beta p_{FD}^j + \gamma p_{DD}^j\nonumber \\&\quad +\varepsilon (\alpha \,\bigl ( \beta p_{FD}^j + ( \mathbf {A}\mathbf {u})_Dp_{DD}^j\bigr ) - \mu p_{SD}^j),\end{aligned}$$

16c $$\begin{aligned} \frac{dp_{FD}^j}{d\tau }&= -b p_S p_{FD}^j +a n p_{SD}^j-\beta p_{FD}^j-\varepsilon \mu p_{FD}^j,\end{aligned}$$

16d $$\begin{aligned} \frac{dp_{DD}^j}{d\tau }&= bp_F p_{SD}^j - \gamma p_{DD}^j+b p_S p_{FD}^j-\varepsilon \mu p_{DD}^j,\end{aligned}$$

16e $$\begin{aligned} \frac{dp_{SH}^j}{d\tau }&= -b p_F p_{SH}^j - a n p_{SH}^j+\beta p_{FH}^j + \gamma p_{DH}^j\nonumber \\&\quad +\varepsilon (\alpha \,\bigl ( \beta p_{FH}^j+ (\mathbf {A}\mathbf {u})_Hp_{DH}^j\bigr ) - \mu p_{SH}^j),\end{aligned}$$

16f $$\begin{aligned} \frac{dp_{FH}^j}{d\tau }&= -b p_S p_{FH}^j + a n p_{SH}^j -\beta p_{FH}^j-\varepsilon \mu p_{FH}^j,\end{aligned}$$

16g $$\begin{aligned} \frac{dp_{DH}^j}{d\tau }&= bp_F p_{SH}^j - \gamma p_{DH}^j+b p_S p_{FH}^j- \varepsilon \mu p_{DH}^j\; \end{aligned}$$

where $$j\in \{r,m\}$$.

Note that a resident individual in $p_{SH}^r+p_{SD}^r$ can encounter a feeding variant individual in $p_{FH}^m+p_{FD}^m$ and both move all to their defending states $p_{DH}^r$, $p_{DD}^r$, $p_{DH}^m$ and $p_{DD}^m$ respectively, and start to fight. The same holds for a searching variant individual which encounters a feeding resident individual. The equations for all sub-populations are now split up into the resident, $p_D^r=p_{DD}^r+p_{DH}^r$, and the variant, $p_D^m=p_{DD}^m+p_{DH}^m$, sub-populations. The trait of the variant equals $\rho ^m=p_{DH}^m/p_D^m$ and then we have $1-\rho ^m=p_{DD}^m/p_D^m$. For the total population we introduce $p_S=p_{S}^r+p_{S}^m$ and $p_F=p_{F}^r+p_{F}^m$ besides $p_D=p_{D}^r+p_{D}^m$. Then the gain $G$ is given by Eq. (14e): $G=2 b p_F p_{S}/p_{D}$.

The average gain of the hawk (resp. dove) equal $(\mathbf {A}\mathbf {u})_H$ (resp. $(\mathbf {A}\mathbf {u})_D$) are the sum of the resident and variant populations. These two expression are using the elements in the pay-off matrix in Eq. (1):

17a $$\begin{aligned} (\mathbf {A}\mathbf {u})_H&= \frac{G-C}{2} \frac{\rho ^rp_D^r+\rho ^mp_D^m}{p_D} + G \frac{(1-\rho ^r)p_{D}^r+(1-\rho ^m)p_{D}^m}{p_D},\end{aligned}$$

17b $$\begin{aligned} (\mathbf {A}\mathbf {u})_D&= \frac{G}{2} \frac{(1-\rho ^r)p_{D}^r+(1-\rho ^m)p_{D}^m}{p_D}. \end{aligned}$$

These expressions are equal for both resident and variant populations. The expressions for the different average gains for the two whole resident and variant populations are

18a $$\begin{aligned} (\mathbf {u}^T\mathbf {A}\mathbf {u})^r&= (\mathbf {A}\mathbf {u})_H\rho ^r+(\mathbf {A}\mathbf {u})_D(1-\rho ^r),\end{aligned}$$

18b $$\begin{aligned} (\mathbf {u}^T\mathbf {A}\mathbf {u})^m&= (\mathbf {A}\mathbf {u})_H\rho ^m+(\mathbf {A}\mathbf {u})_D(1-\rho ^m). \end{aligned}$$

They show the dependency of the two trait values $\rho ^r$ and $\rho ^m$.

The dynamics of the three sub-populations $\{S,F,D\}$ each can be reduced to the following set by adding the hawks and doves versions:

19a $$\begin{aligned} \frac{dn}{d\tau }&=\varepsilon \bigl ( rn(1-\frac{n}{K}) - anp_S^r- an p_S^m\bigr ),\end{aligned}$$

19b $$\begin{aligned} \frac{dp_{S}^j}{d\tau }&= -b p_F p_{S}^j-a n p_{S}^j+\beta p_{F}^j +\gamma p_{D}^j +\varepsilon (\alpha \,\bigl ( \beta p_{F}^j + (\mathbf {u}^T \mathbf {A}\mathbf {u})^j p_{D}^j\bigr ) - \mu p_{S}^j),\end{aligned}$$

19c $$\begin{aligned} \frac{dp_{F}^j}{d\tau }&= -b p_S p_{F}^j + a n p_{S}^j -\beta p_{F}^j-\varepsilon \mu p_{F}^j,\end{aligned}$$

19d $$\begin{aligned} \frac{dp_{D}^j}{d\tau }&= b p_F p_{S}^j- \gamma p_{D}^j + b p_S p_{F}^j -\varepsilon \mu p_{D}^j, \end{aligned}$$

for the resident population, $j=r$, and the variant population, $j=m$.

In order to find the singular strategy $\textsc {ss}$ we are looking for transcritical bifurcations where both trait values $\rho ^r$ and $\rho ^m$ as free parameters. The resulting bifurcation diagram is the pip-plot. It is a convenient graphical tool to study the adaptive dynamics of a trait. In the next two sub-sections we will analyse the equilibrium and limit cycle case.

#### Trait dynamics analysis: equilibrium

We start with the analysis of the AD-model Eqs. (19) using symbolic analysis and with the computer package (Maple 2008). We are interested in the equilibrium values obtained by solving the set of equation where the right-hand sides of system (16) are zero. We are especially interested in the stability of the boundary equilibrium where the variant population is extinct.

The non-zero elements in the Jacobian matrix evaluated at the equilibrium where the variant population is absent are indicated in:20The matrix has the following shape

21 $$\begin{aligned} \mathbf {J}= \left( \begin{array}{ll} \mathbf {J}_{44}&{}\quad *\\ \mathbf {0} &{}\quad \mathbf {J}_{33} \end{array}\right) . \end{aligned}$$

We are interested in the rate of invasion of the variant population into the environment where the resident population exists stably. We define the “invasion fitness” denoted by $s_e(\rho ^r,\rho ^m)$ now as follows:

22 $$\begin{aligned} s_e(\rho ^r,\rho ^m)= \lambda _1, \end{aligned}$$

where $\lambda _1$ is the real dominant eigenvalue of the Jacobian matrix $\mathbf {J}$ evaluated at the boundary equilibrium where the variant population is absent.

The ss is now fixed by the two equations:

23 $$\begin{aligned} s_e(\rho ^r,\rho ^m)|_{p_S^m=p_{F}^m=p_{D}^m=0}=0,\end{aligned}$$

24 $$\begin{aligned} s_e(\rho ^r,\rho ^m)|_{p_S^r=p_{F}^r=p_{D}^r=0}=0, \end{aligned}$$

where both equities fix a codimension-one transcritical bifurcation curve in the bifurcation diagram $(\rho ^r,\rho ^m)$ and hence the ss is the intersection of these two curves.

We can use the special shape of the Jacobian matrix to get analytical expressions for the solution of these equation. We start with the calculation of $\det \mathbf {J}$ which equals the product of all eigenvalues. Linear Algebra learns that for matrices given in Eq. (21) we have $\det \mathbf {J}=\det \mathbf {J}_{44} \times \det \mathbf {J}_{33}$. We know that $\det \mathbf {J}_{44} \ne 0$ for parameters in the region where the equilibrium is stable (eigenvalues are strictly negative or have strictly negative real parts). Hence, we are looking for parameters where

$$\begin{aligned} \det \mathbf {J}_{33}=0. \end{aligned}$$

Using Maple (2008) we found the following expression for $\rho ^r$ as the solution of this equation

25 $$\begin{aligned} \rho ^r=\frac{\gamma +\varepsilon \mu }{C}, \end{aligned}$$

for all $0 \le \rho ^m \le 1$. It is important to notice that the right-hand side does not depend on $\rho ^m$ the second bifurcation parameter and hence the $\textsc {ss}$ occurs at $\rho ^*=\rho ^m=\rho ^r$. Furthermore this shows that the $\textsc {ss}$ does not depend on the carrying capacity $K$.

**Fig. 6:**
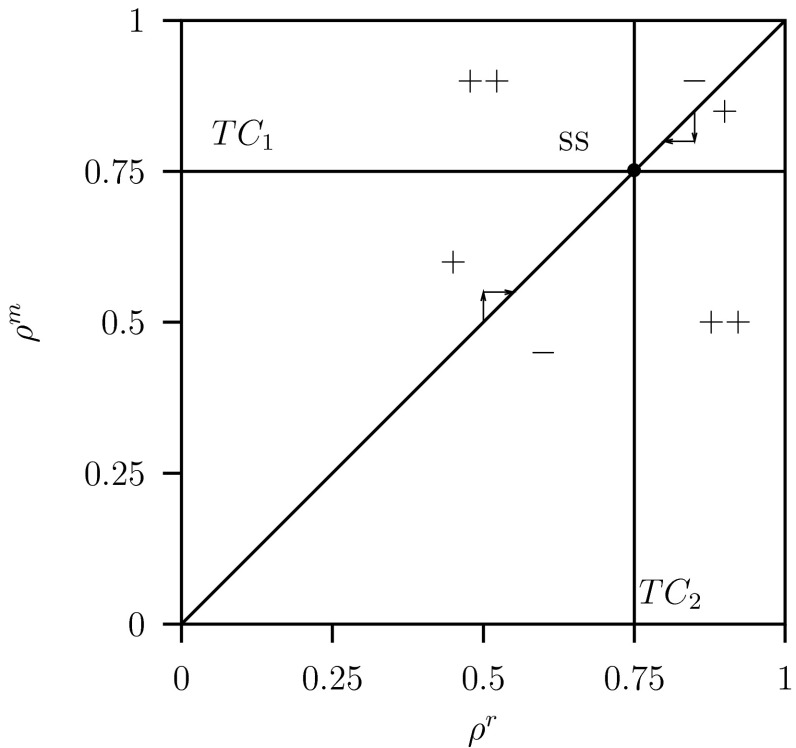
Pairwise invasibility plot (pip) for system Eq. (19) plotting the invasion fitness of a variant population with trait value $\rho ^m$ invading a resident population with trait value $\rho ^r$. The *curves* $TC_1$ and $TC_2$ are transcritical bifurcation for equilibria curves which are *vertical* and *horizontal straight lines*. Point $\textsc {ss}$ (a neutral stable strategy, nss) and is the intersection of these two lines at $\rho ^r=\rho ^m=G/C=0.75$. The arrows illustrate an invasion step to wards the $\textsc {ss}$. Parameter values as given in Table 1 and $C=2$, $K=2$

In order to explain these results we show the pip-plot in Fig. 6 for $C=2$, $K=2$ and the other parameter values given in Table 1. In this figure the transcritical curve $TC_2$ is a vertical line where $\rho ^r_{TC}=\rho ^*=0.75$ given by Eq. (25). Using mutual invasibility of the variant population by the resident population gives also the horizontal curve $TC_1$. The intersection at the diagonal is the $\textsc {ss}$. The arrows show the dynamics of the trait by a step-wise change along the diagonal toward the $\textsc {ss}$. We assume that each step is an elementary step in a learning process in which the fractions of the hawks and doves in the defending population adapt. The convergence along the diagonal in the pip-plot is assumed fast because the adaption steps are assumed to be small while the replacement of the resident population by the variant population due to learning is assumed to be very fast. This is related to the assumption that the intra-specific fighting between the predators occurs at a fast time scale where $c=\infty$ in the RD approach.

Observe that invasion of the variant population is related to the zero eigenvalue on the $TC_2$ curve where $p_S^m=p_F^m=p_D^m=0$. Hence, the boundary space where the variant invades is three dimensional and not one, as expected for the invasion of one predator population. The interpretation of the rate of invasion is now related to the growth rates of the three compartments where both hawks and doves in each of these compartments are invading whereby their ratio is prescribed by the trait-value $\rho ^m$.

The parameter space $(\rho ^r,\rho ^m)$ is divided in three different regions: ‘$+$’-the variant can invade and replaces the resident, ‘$-$’-the variant cannot invade and goes extinct, ‘$++$’-the variant can invade but cannot replace the resident which leads to coexistence. However, the transcritical bifurcations that separate these different regions are vertical and horizontal lines and therefore the curves in Fig. 6 show that the $\textsc {ss}$ is neutral stable (see also Geritz et al. 1998, Fig. 9b). When in the learning process the trails are myopic the steps along the diagonal become smaller and smaller when approaching the $\textsc {nss}$ point, and it will effectively be a $\textsc {css}$ point and an end-point. When on the other hand these steps are random and finite, the system enters the coexistence region ‘$++$’ and the population becomes a coalition and the $\textsc {nss}$ point on the diagonal is not the end-point. Then we have to consider the invasion of this coalition of two resident populations by a variant population. By simulation we found that introduction of a variant population with arbitrarily trait value, leads to convergence to a new interior point where all compartments are positive but this equilibrium point is neutral stable (that is one eigenvalue is zero). When invasion of this coalition between three populations by a next variant with an arbitrarily trait value we obtained the same result, now four predator populations can coexist, and again the equilibrium is neutral but now two eigenvalues are zero. These results suggest that the nss is invasible by new variants. However, when the next variant population lies in the ’$+$’-region of the pip-plot, then this variant will out-compete all the other resident populations and the whole process of invasion, replacement, coexistence and so on, starts all over again. This shows that there will be episodes with coalition between many populations with different trait values, but the varying trait values will stay close to the $\textsc {nss}$ point.

**Fig. 7:**
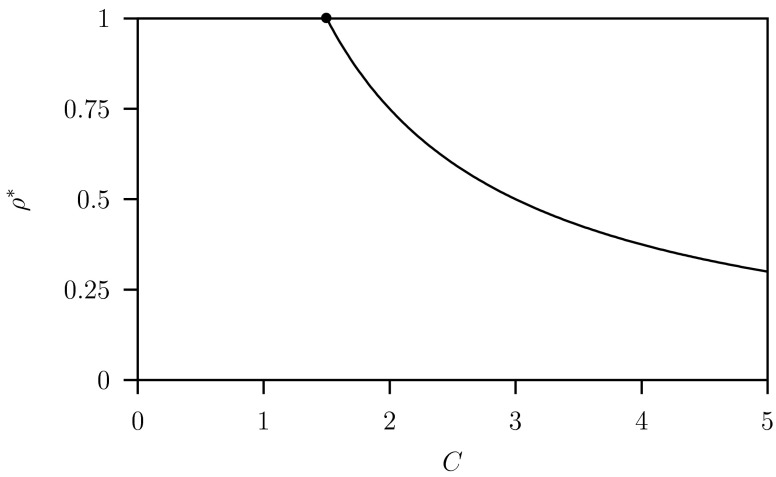
Two-parameter diagram $C$ versus $\rho ^r$ for system Eq. (19). This is the approximation of the codimension-two $\textsc {ss}$ point as the intersection point of the two transcritical bifurcation lines $TC_1$ and $TC_2$ shown in Fig. 6 starting for $C=\gamma +\varepsilon \mu =1.5$ at $\rho ^*=G/C$ for $C=2$. The analytical expression for the curve is $\min (1,G/C)$ where $G$ is given in Eq. (14e). Parameter values as given in Table 1 with $K=2$

In Fig. 7 we plotted the $\rho ^*$-value for the $\textsc {ss}$ as a function of the costs $C$ for $K=2$ where the system is in equilibrium. First the transcritical bifurcation $TC_2$ with $\rho ^r$ as bifurcation parameter and simultaneously varying $\rho ^m=\rho ^r-\delta$ was found (see Fig. 6 with $C=2$ and $K=2$). Here $\delta$ is a small, arbitrarily numerical parameter. Then we continued this transcritical bifurcation where $p_S^m=p_F^m=p_D^m=0$ with $C$ and $\rho ^r$ and $\rho ^m=\rho ^r-\delta$ the two varying parameters starting from $C=2$ while $K=2$ fixed. With this $C$-value we have $\rho ^*<1$, so the dimorphic case with hawks and doves. Lowering $C$, $\rho ^*$ becomes $1$ at $C=\gamma +\varepsilon \mu =1.5$ where we have the switch to the monomorphic case with only hawks. Numerically we found that this curve for all $C$ is indeed close to the graph of the relationship $\rho ^*=G/C$ where $G$ is given in Eq. (14e).

These results hold only when the long-term dynamics of the predator–prey system is a stable equilibrium. In the next section we discuss the case where the long-term dynamics is a limit cycle.

#### Trait dynamics analysis: limit cycle

We continue with the analysis of the limit cycle case with the example where the parameter values are $C=2$ and $K=20$ instead of $K=2$ as in the previous section. Two of the four eigenvalues of matrix $\mathbf {J}_{44}$ in Eq. (21) are complex conjugated with positive real parts and therefore the equilibrium of the resident population is unstable. Numerically we found a stable limit cycle (periodic solution) that originates from a Hopf bifurcation that is passed when $K$ is increased from $K=2$ to $K=20$ while $C=2$.

The trait is now the invasion rate $s_c(\rho ^r,\rho ^m)$ of the rare variant population via a boundary limit cycle with period $T_0$:

26 $$\begin{aligned} s_c(\rho ^r,\rho ^m) = \log (\mu _1), \end{aligned}$$

where $\mu _1$ is the magnitude of the dominant Floquet multiplier evaluated at the periodic solution of the resident system where the size of the variant population is zero: $p_S^m(t)=p_F^m(t)=p_D^m(t)=0$ for $0\le t\le T_0$ (see also Metz et al. 1992). The biological interpretation of $s_c(\rho ^r,\rho ^m)$ is the average per capita growth rate over one cycle. In the case of limit cycles no analytical results are available as in the equilibrium case. However, by replacing eigenvalues by multipliers with their correct interpretation we can also calculate the transcritical bifurcation curves in a pip-plot using the numerical bifurcation analysis software auto (Doedel and Oldeman 2009).

**Fig. 8:**
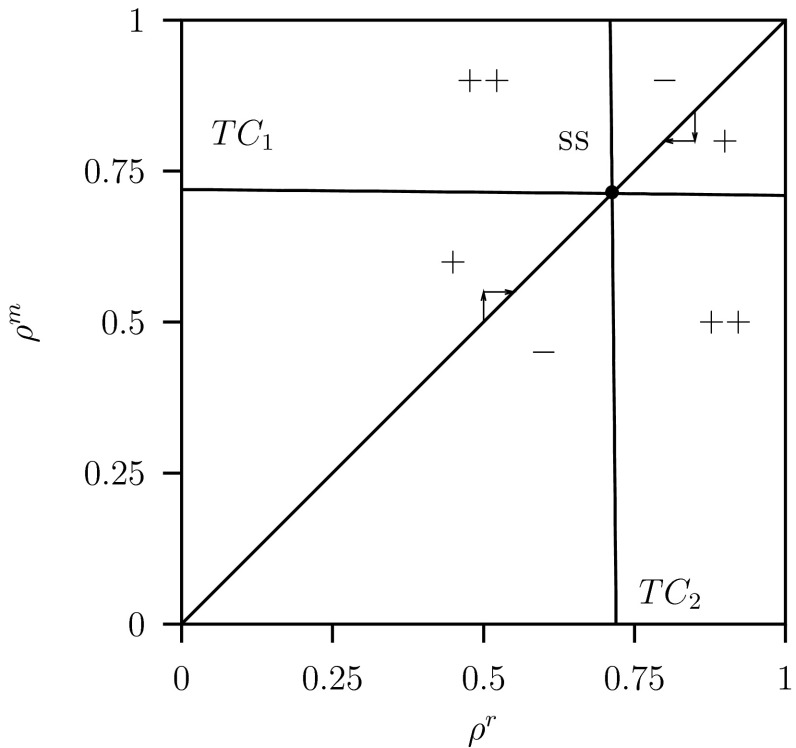
Pairwise invasibility plot (pip) for system Eq. (19) plotting the invasion fitness for a variant with trait value $\rho ^m$ invading a resident population with trait value $\rho ^r$. The curves $TC_1$ and $TC_2$ are transcritical bifurcation for limit cycles curves which are now not straight lines as was the case for equilibria given in Fig. 6. Point $\textsc {ss}$ (a continuously stable strategy, css) and is the intersection of these two curves. The *arrows* illustrate an invasion step toward the $\textsc {ss}$. The convergence along the diagonal in the pip-plot is assumed to be fast. Parameter values as given in Table 1 and $C=2$, $K=20$

The resulting pairwise invasion pip-plot is shown in Fig. 8 for the parameter values $C=2$ and $K=20$. The transcritical bifurcations $TC_j$ are the zero fitness isoclines (solid lines) now for limit cycles. The intersection of the diagonal with the other two curves $TC_j$, $j=1,2$, corresponds to a $\textsc {ss}$. The parameter space $(\rho ^r,\rho ^m)$ is again divided in three different regions: ‘$+$’-the variant population can invade and replaces the resident, ‘$-$’-the variant cannot invade and goes extinct, ‘$++$’-the variant population can invade but cannot replace the resident population which leads to coexistence, a coalition between the resident and the variant population. From the transcritical bifurcation curves in Fig. 8 we conclude that there is a $\textsc {css}$ (compare with Geritz et al. 1998, Fig. 4 case c). For, these transcritical bifurcations for limit cycles are not vertical and horizontal as in the case of the transcritical bifurcation curves of the equilibria in Fig. 6 where $C=2$ and $K=2$. Therefore this $\textsc {ss}$ is an end-point, that is: the resident population is non-invasible by any variant population. The arrows show the dynamics of the trait by a step-wise change along the diagonal towards the $\textsc {ss}$ point and close to the $\textsc {ss}$ coexistence never occurs.

**Fig. 9:**
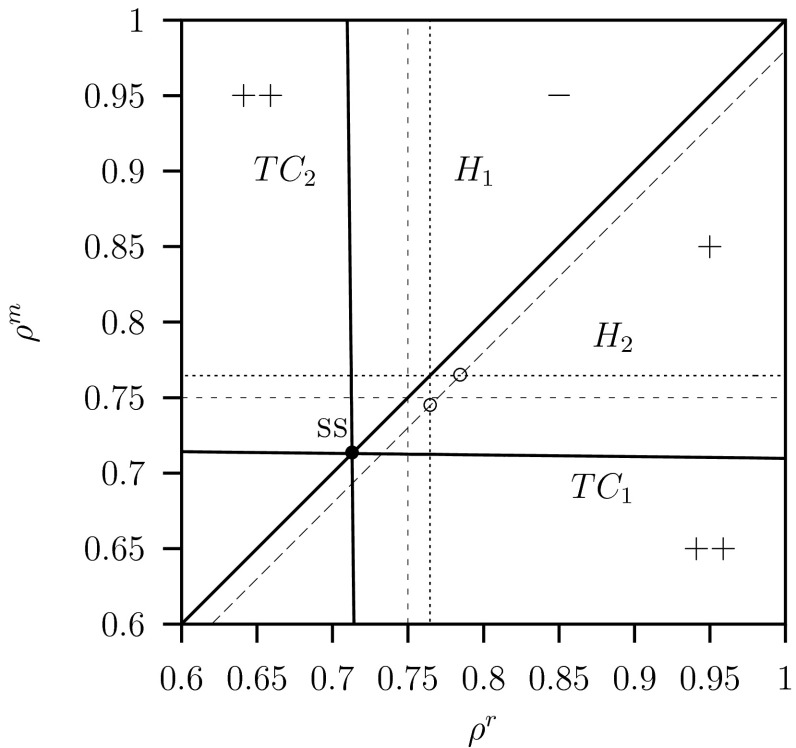
Detail of Fig. 8 the pairwise invasibility plot (pip) for system Eq. (19). Also the two Hopf bifurcations curves (*dotted lines*) $H_1$ and $H_2$ are depicted. Furthermore the line $\rho ^m=\rho ^r- \delta$ (*dashed line*) is shown. Along this line in the interval in the $++$ region there is coexistence. Parameter values as given in Table 1 and $C=2$, $K=20$

The region close to the $\textsc {ss}$ is shown enlarged in Fig. 9 where also the curves for the unstable equilibrium are shown. The vertical and horizontal transcritical bifurcation curves for the equilibria intersect at the unstable equilibrium $\textsc {ss}$ point. This occurs at the value $\rho ^r =\rho ^m=\rho ^*=0.75$ given by Eq. (25). This pattern is the same as that for the stable equilibria given in Fig. 6 for $C=2$ and $K=2$.

Furthermore two Hopf bifurcations $H_1$ and $H_2$ are shown. At these points the boundary equilibrium becomes unstable, at $H_1$ the equilibrium where the variant population is extinct and at $H_2$ where the resident population is extinct.

**Fig. 10:**
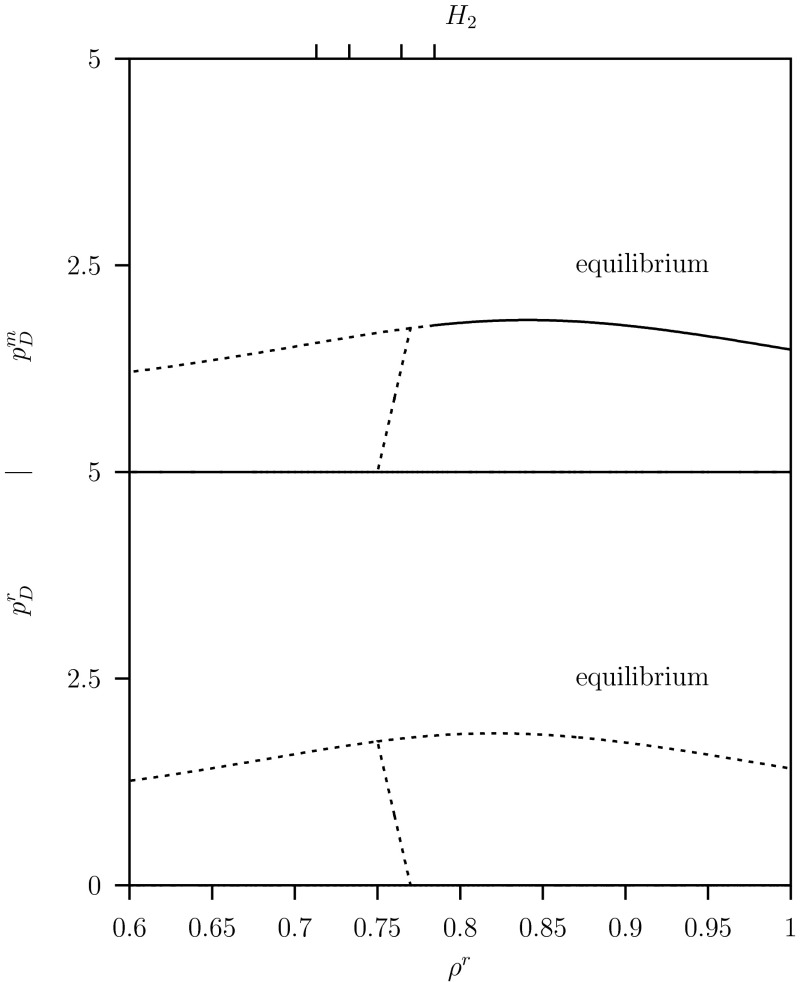
One-parameter diagram for the resident population $p_D^r$ and variant population $p_D^m$, where $\rho ^r$ varies while $\rho ^m=\rho ^r- \delta$ for AD approach system Eq. (19). See Fig. 9 for the *pip-plot with the line* $\rho ^m=\rho ^r- \delta$. Only results for the system in equilibrium are shown. *Dashed curves* show either unstable equilibrium. *Solid curve* above $H$ indicates a stable equilibrium, where $p_D^r=0$, that is for the variant population only. Parameter values as given in Table 1 where $C=2.0$ and $K=20$

We show in Fig. 10 for $C=2$ and $K=20$ in a one-parameter diagram for the equilibria where $\rho ^r$ varies along the sub-diagonal line $\rho ^m=\rho ^r- \delta$ in the pip-plot depicted in Fig. 9, the size of the resident, $p_D^r$, and the variant defending predator populations, $p_D^m$. There is only (unstable) coexistence in the small interval $0.75 \le \rho ^r \le 0.775$ above the unstable ss point at $\rho ^*=0.75$.

**Fig. 11:**
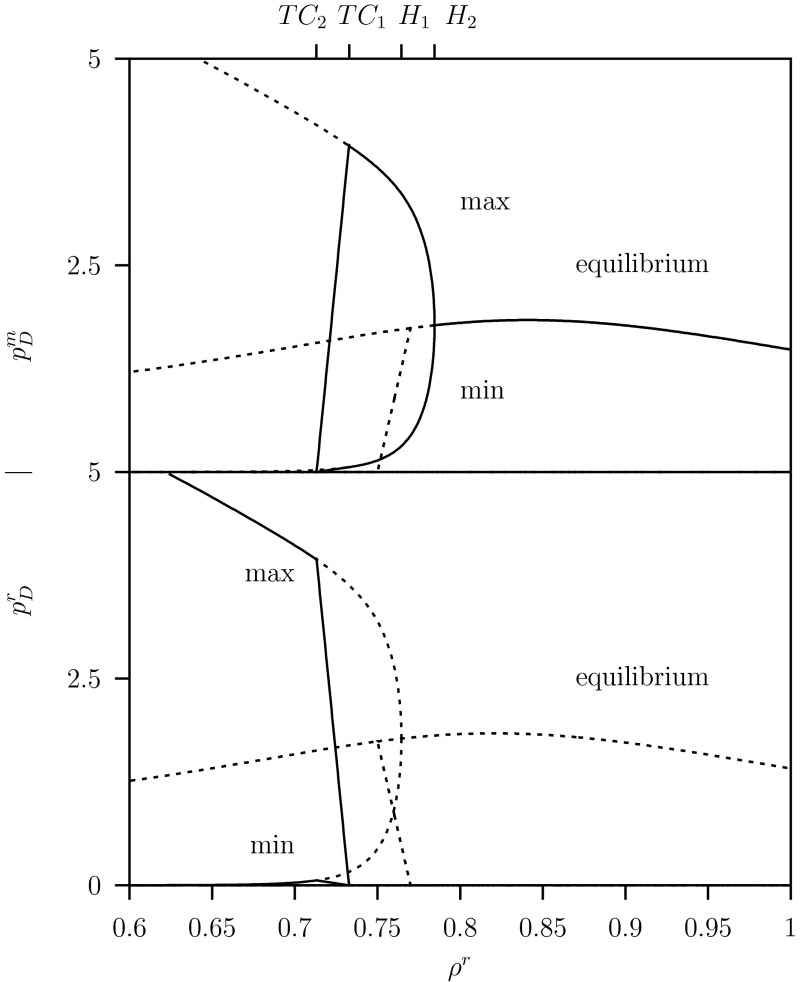
One-parameter diagram for the resident population $p_D^r$ and variant population $p_D^m$, where $\rho ^r$ varies while $\rho ^m=\rho ^r- \delta$ for system Eq. (19). See Fig. 9 for the *pip-plot with the line* $\rho ^m=\rho ^r- \delta$. Now besides the equilibrium results the limit cycle results are shown. *Solid* (*almost straight*) *curves* between $TC_1$ and $TC_2$ are maximums and minimums of the stable limit cycle where both resident and variant populations coexist. Between $TC_1$ and $H$ there is a limit cycle and above $H$ a stable equilibrium, where $p_D^r=0$, that is for the variant population only. Below $TC_2$, we have $p_D^m=0$ where the resident population exist stably. *Dashed curves* show either unstable equilibrium or maximums and minimums of unstable limit cycles. Parameter values as given in Table 1 where $C=2.0$ and $K=20$

In Fig. 11 for the same parameter values $C=2$ and $K=20$, we show the one-parameter diagram for the limit cycles. Here the maximum and minimum values are plotted in the regions where the system oscillates periodically below the Hopf bifurcations $H_1$ and $H_2$.

Above the transcritical bifurcation point $TC_1$ in the ‘$+$’-region: the variant can invade and replaces the resident. There the resident population is extinct $p_D^r=0$. Below the Hopf bifurcation $H_2$ the variant population is unstable and the population oscillates. In the ‘$++$’-region between the two transcritical bifurcation points $TC_1$ and $TC_2$, $j=1,2$ the variant can invade but cannot replace the resident which leads to coexistence where both populations oscillate. In the region below $TC_2$, in the ‘$-$’-region, the variant population cannot invade and is extinct while the resident population oscillates, see lower panel in Fig. 11.

The description of the singular strategy ss trait values in this section shows that when the predator–prey system possesses a stable equilibrium the ss trait value is simply given by the expression given in Eq. (25) as a function of the parameter values $\gamma ,\varepsilon ,\mu$ and $C$ (and hence independent of $K$). When the predator–prey system possesses a stable limit cycle on the other hand, the ss trait value has to be determined numerically. This will be explained in the next section.

### AD-results

**Fig. 12:**
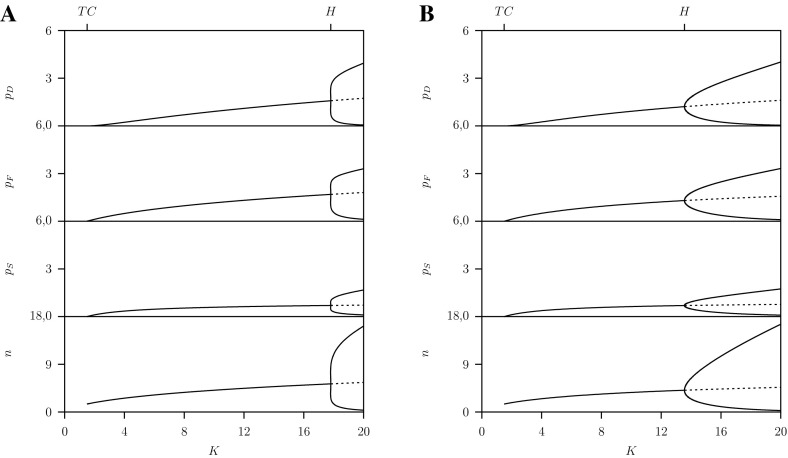
One-parameter diagrams for the densities as function of the carrying capacity $K$ predicted by the AD approach, system Eq. (19). Below $TC$, all predator sub-populations are zero the population is extinct. Between $TC$ and Hopf bifurcation point $H$ a stable equilibrium and above $H$ there is a limit cycle

Figure 12a gives the one-parameter diagram with the prey’s carrying capacity $K$ as free parameter for the densities $n, p_S, p_F, p_D$ where again $C=2$. Here we started on the transcritical bifurcation $TC_2$ at $\rho ^m=\rho ^*-\delta$, so a point very close to the $\textsc {ss}$ point, and continued this transcritical bifurcation $TC_2$ with $\rho$ and $K$ as free parameters. For decreasing $K$ at the transcritical bifurcation $TC$ the predator population goes extinct whereby all three sub-populations disappear simultaneously. For values above the Hopf bifurcation $H$ where the equilibrium is unstable we continued the transcritical bifurcation of the limit cycle as explained in the previous section.

Similar results are shown in Fig. 12b now for $C=1$ in the monomorphic region where $G>C$. Consequently, for all $K$ the $\textsc {ss}$ is the right-top corner of the pip-plot. In these pip-plots the diagonal divides the plot into two halves, below the diagonal there is the ‘$-$’-region where always the resident wins and above the diagonal the ‘$+$’-region where the variant population always wins. This show directly that the $\textsc {ss}$ with $\rho ^*=1$ is a $\textsc {css}$ and also the end-point (for comparison see Fig. 6 where $C=2$ and $K=20$). Notice that this also holds for the $K$-region where the equilibrium is unstable and a limit cycle exists (see Fig. 12b) with small amplitude just above the Hopf bifurcation.

Now we are interested in the transition from the stable equilibrium to the stable limit cycle between $K=2$ and $K=20$ when $C=2$. Figure 11 indicates that we are looking for the $K$ value where all four bifurcation points coincide.

**Fig. 13:**
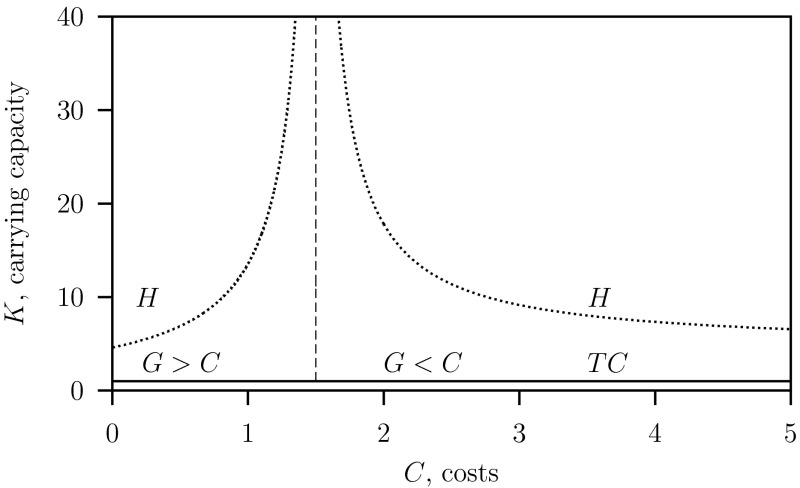
Two-parameter bifurcation diagram $C$ versus $K$ for the AD approach system Eq. (19) where $\varepsilon = 1$ with $G$ given in Eq. (14e). Parameter values as given in Table 1 and see Fig. 2 for an explanation of the symbols

A method to continue the Hopf bifurcation in the parameter bifurcation diagrams where $C$ and $K$ are the free parameters and using auto (Doedel and Oldeman 2009) is as follows. In this special case we know that singular strategy trait value, given a $C$ value and independent of $K$, is given by Eq. (25). This value can be substituted directly in system Eq. (14) where $\rho ^r=\rho ^m=\rho ^*$ and using Eq. (8) it is possible to continue the Hopf bifurcation in the two parameter space $(C,K)$. In order to avoid numerical problems we take an approximated value: $\rho ^r=\rho ^*+\delta$ and $\rho ^m=\rho ^*-\delta$, so coexistence points for the resident and variant populations but close to the $\textsc {ss}$ point. The resulting Hopf bifurcation curves for the mono- and dimorphic cases are shown in the two-parameter bifurcation diagram Fig. 13.

In the next Sect. 4 we will compare the results given in Figs. 12 and 13 with those for the RD approach given in Figs. 3 and 4.

## Discussion and conclusions

In a previous paper by Auger et al. (2006) we studied the dynamical behaviour of a predator–prey system where the predators feed on the prey via direct consumption and also via fighting. The outcome of the fighting was modelled as a continuous-trait hawk–dove game where the defending predator individuals play either the hawk or the dove tactic with gain and costs as defined in the classical hawk–dove game pay-off matrix Eq. (1). The predator individuals go through a sequence of behavioral stages: searching and feeding stages and when a feeding individual encounters a searching individual they enter the third defenders stage. In this stage they can either keep the prey, share the prey or lose it, while playing hawk or dove tactics.

The individual behavioral processes run often at a much faster time scale than those of the population size changes. Therefore in Auger et al. (2006) we applied a time scale separation technique to reduce the dimension from six for the predator plus one for the size of the prey population, to two, namely for the sizes of the prey and predator populations. Crucial was the fact that the hawk–dove game occurs at the fast time scale, often called the quasi-steady state assumption. As a consequence the gain and also the trait of the hawk–dove game are density-independent, see Eq. (27c) in the Appendix. This is corrected in this paper by using Eq. (14e) or when applicable Eq. (15) to describe the gain. Then, in the full model the gain and consequently also the trait of the predator population, are density-dependent, and this is essential when the predator–prey system oscillates periodically.

Hence, in the RD-approach through changing tactics of a variant individual with a gain above average population gain, the strategy of the predator population is continuously and instantaneously adapted to the momentary ess trait value given as the actual equilibrium of the replicator equation Eq. (11). The governing equations are all ode’s. To describe the dynamics of the trait either the replicator equation, which is itself an ode, or directly the quasi-static ess trait value, yielding an algebraic equation, is used. Hence, the same type of bifurcation analysis can be done as by Auger et al. (2006).

In this paper we modelled, besides the RD-approach also the AD-approach whereby competition shapes the dynamics via possible invasion of the rare variant population into the resident population. In the AD-approach applied to eco-evolutionary problems, the dynamics of the trait and the full evolutionary trajectory of the population are generally described by the canonical equation, see (Dieckmann and Law 1996; Geritz et al. 1998; Dercole et al. 2003; Dercole and Rinaldi 2008). The parameters in these canonical equations describing the trait dynamics, are directly related to inheritance of traits via mutations. A zero invasion fitness gradient determines the singular strategy state. Here we use a time-scale separation argument and no alternative expressions for parameters of such a canonical equation related to learning are needed. Then in terms of bifurcation theory the traits of the resident and variant populations are bifurcation parameters and the ss point is fixed by the intersection of two transcritical bifurcations. This process is graphically illustrated in pip-plots. Here also a Hopf bifurcation is involved, namely when the predator–prey system starts to cycle under nutrient enrichment.

In the literature the AD-approach has often been applied to simple ’toy’ models to get theoretical insight. In Dieckmann and Metz (2006) this was the case where they studied the hawk–dove game embedded in a simple population dynamical one-dimensional discrete-time model with non-overlapping generation. In that paper variations of this model were used to find conditions for having degeneracy, namely that the ss is neutral (nss): a general result of game theory, widely known as the *Bishop–Cannings theorem* (Bishop and Cannings 1978; Geritz et al. 1998; Dieckmann and Metz 2006). They showed that reward fluctuations between generations removes already the degeneracy. This is similar to our finding that when the dimorphic predator–prey system is oscillating the ss is continuously stable (css). The use of (numerical) bifurcation analysis techniques makes it possible to analyse more complex and biologically more realistic models such as the continuous-time predator–prey system.

In the RD-approach two-parameter $(C,K)$ bifurcation diagrams for the reduced system Eq. (28) described in the Appendix and the four dimensional system Eq. (14) are given in Fig. 2 ($\varepsilon =0$) and Figs. 3 ($\varepsilon =1$) whereby in both models $c=\infty$. In these models the regimes where the monomorphic and the dimorphic systems occur are separated by the vertical line at $C=\gamma +\varepsilon \mu$. This explains the shift in this vertical line from $C=1$ (where $\varepsilon =0$), for the reduced model to $C=1.5$ for the full model (where $\varepsilon =1$).

Since the transcritical bifurcation $TC$ is an equilibrium bifurcation and because in equilibrium the RD- and AD-approach give the same results, the $TC$-curves in the two-parameter $(C,K)$ bifurcation diagrams are the same for both game dynamics formulations.

From Figs. 4 versus 12 and Figs. 3 versus 13 we conclude that for the two RD- and AD-formulations the same solutions are predicted in stable equilibrium regions of the two-parameter bifurcation diagram. There are differences in the monomorphic case, $G>C$, when the amplitude of the limit cycles are large so that switching between monomorphic and dimorphic populations occurs in the RD-approach only. Furthermore both approaches give quantitatively different results in the dimorphic case, $G>C$, above the Hopf bifurcation where a stable limit cycle for the predator–prey system exists.

In both approaches, the monomorphic and the dimorphic cases are separated by the vertical curve at the same $C=1.5$. On the left-hand side of the vertical curve where $G>C$ the population is monomorphic where although the gain $G$ is density-dependent, the trait $\rho ^*=1$ is density-independent. On the right-hand side of the vertical curve where $G<C$ there is only equality when the predator–prey system in both models are in stable equilibrium with $\rho ^*=G/C$. We conclude that the bifurcation pattern is qualitatively the same for all two-parameter $(C,K)$ bifurcation diagrams predicted by the different models. Two of these models are for $\varepsilon =0$, Fig. 2, and $\varepsilon =1$, Fig. 3, and this indicates that the results are robust for the parameter values given in Table 1 for intermediate values where $\varepsilon \in [0,1]$. In the $C$-parameter range round the threshold where costs equal gain and the population changes from mono- to dimorphic, all models predict that there is equilibrium for arbitrary prey’s carrying capacities and that the “paradox of enrichment” phenomenon does not occur.

While the predicted trait value is an ess as equilibrium of the replicator equation in the RD-approach, the ss in the AD-approach is a nss. New variant populations can coexist with the resident populations forming a stable coalition between two populations with a different trait value. This shows that the introduction of a behavioural process for the predator population makes it possible to have two different predator populations, namely with different trait values, living on one prey population. This is in contradiction with the “competitive exclusion” principle by Hardin (1960). Obviously the introduction of a behavioural process (here with two states hawks and doves) facilitates the stable coexistence of two populations (called supersaturation).

We presume that the RD-approach is consistent with individual learning (IL). Individuals in a replicator system have localized knowledge concerning the system as a whole (Gintis 2000). On the other hand in the AD-approach the invasion fitness is determined by competition between two populations where in each population the individuals have the same trait value. Then effectively, the population does adopt a best reply to the overall trait value which is consistent with social learning (SL).

When variant populations differ randomly from the resident with myopic learning by trail and error, then for stable equilibria of AD-approach system the trait values remain in general close to the predicted nss value. Notice that this degeneracy, also discussed by Dieckmann and Metz (2006), exists in equilibrium even though feedback from the environment via the ecological processes such a searching for prey and feeding, are taken into account. However, when the systems are periodically oscillating in the AD approach the ss is not degenerated, it is a css. Hence for this predator–prey system oscillatory dynamics does not facilitate coexistence of two predator populations living on a single prey population.

These results illustrate how intra-specific trait dynamics matters in predator–prey interaction and therefore also in ecosystem dynamics in general.

## Appendix: Time scale separation reduction

We recall shortly some of the results already obtained by Auger et al. (2006). The full model is described by the governing seven dimensional set of equations Eqs. (14) with $\varepsilon =1$ and $\tau =t$. In Auger et al. (2006) this model was reduced by assuming $c=\infty$ and $\varepsilon =0$. Because the game dynamics described by the RD-approach is assumed to be fast, it is separated from the ecological searching and feeding dynamics. Therefore we can use replicator game equilibria of Eq. (11) given in Eq. (12) in Eqs. (7, 8). Furthermore with $\varepsilon =0$, also the ecological searching and feeding processes are assumed to be fast with respect to the other ecological processes such as prey growth and predator mortality. This is similar to the assumptions underlying the derivation of the Holling-disk equation.

At this RD equilibrium the average gain for the whole population Eq. (8) and using Eq. (11) reads:

27a $$\begin{aligned} \mathbf {u}^{*T}\mathbf {A}\mathbf {u}^*&=\left\{ \begin{array}{ll} \frac{G}{2}-\rho ^{*2} \frac{C}{2}=\frac{G}{2}(1-\frac{G}{C}) &{}\quad \text {if}\, G<C \\ \frac{G}{2}- \frac{C}{2}=\frac{G}{2}(1-\frac{C}{G}) &{} \quad \text {if}\, G>C, \end{array} \right. \end{aligned}$$

where Eq. (12) becomes

27b $$\begin{aligned} \rho ^*=\frac{p_{DH}}{p_{D}}=\frac{G }{C},\quad 1-\rho ^*=\frac{ p_{DD}}{p_{D}}=\frac{C-G }{C}. \end{aligned}$$

This means that the strategy and trait value is continuously adapted instantaneously also when the system is periodically oscillating.

Because $\varepsilon =0$ and $c=\infty$, in predator–prey equilibrium Eq. (14d) yields

27c $$\begin{aligned} G^*=\gamma , \end{aligned}$$

where $\gamma$ is a parameter (see Table 1).

In Auger et al. (2006) using again $\varepsilon =0$, explicit expressions for the quasi-equilibrium values for the total searchers $p_S^*$, feeders $p_F^*$ and defenders $p_D^*$ are derived.

The reduced model reads

28a $$\begin{aligned} \frac{dn}{dt}&=rn\left( 1-\frac{n}{K}\right) -a n p_{S}^{*}, \end{aligned}$$

28b $$\begin{aligned} \frac{dp}{dt}&=\left\{ \begin{array}{ll} \alpha \big (\beta p_F^*+ \frac{G}{2} (1-\frac{G}{C})p_{D}^{*} \big )-\mu p &{}\quad \text {if}\, G<C \\ \alpha \big (\beta p_F^*+ \frac{G}{2} (1-\frac{C}{G})p_{D}^{*}\big )-\mu p &{}\quad \text {if}\, G>C, \end{array} \right. \end{aligned}$$

where the values of $p_{S}^{*}$, $p_{F}^{*}$, $p_{D}^{*}$ are given by

28c $$\begin{aligned} p_S^*&= \frac{\gamma (b p-\beta -a n) + \sqrt{\gamma ^2 (b p-\beta - an)^2+ 8ab\gamma \beta np+4\gamma ^2 \beta b p}}{2(2a b n+b\gamma )},\end{aligned}$$

28d $$\begin{aligned} p_F^*&= \frac{an}{\beta +b p_S^*}p_S^*,\end{aligned}$$

28e $$\begin{aligned} p_{D}^{*}&=p-p_{S}^{*}-p_{F}^{*}. \end{aligned}$$

The switch between the models when $G =C$ is smooth with respect to the state variables $n(t)$ and $p(t)$.

Thereafter the six quasi-steady state solutions for $p_{SD}^*,p_{FD}^*,p_{DD}^*$ for the dove predators and $p_{SH}^*,p_{FH}^*,p_{DH}^*$ for the hawk predators are expressed in the slow state variables $n(t)$ and $p(t)$. The quasi-steady state values of these variables can be calculated with the next formulas:

28f $$\begin{aligned} p_{FD}^*&= \frac{an\gamma }{(b p_S^*+\beta ) (bp_F^* + a n )- an\beta }p_{DD}^*,\end{aligned}$$

28g $$\begin{aligned} p_{SD}^*&= \frac{\beta p_{FD}^* + \gamma p_{DD}^*}{b p_F^* + a n},\end{aligned}$$

28h $$\begin{aligned} p_{DD}^*&=(1-\rho ^*)p_{D}^{*}.\end{aligned}$$

28i $$\begin{aligned} p_{FH}^*&=\frac{an\gamma }{(b p_S^*+\beta ) (bp_F^* + a n )- an\beta }p_{DH}^*,\end{aligned}$$

28j $$\begin{aligned} p_{SH}^*&=\frac{\beta p_{FH}^* + \gamma p_{DH}^*}{b p_F^* + a n},\end{aligned}$$

28k $$\begin{aligned} p_{DH}^*&=\rho ^*p_{D}^{*}. \end{aligned}$$

The proportions of hawk and dove individuals divided in each population stages (searchers, feeders and defenders) are equal. For the defenders we have Eq. (27b) giving $\rho ^*/(1-\rho ^*)=p_{DH}^*/p_{DD}^*$. Then the expression for the other sub-populations we have $\rho ^*/(1-\rho ^*)=p_{SH}^*/p_{SD}^*=p_{FH}^*/p_{FD}^*$. This also holds for the total density of hawks ($p_\mathcal{H}^*=p_{SH}^*+p_{FH}^*+p_{DH}^*$) versus doves ($p_\mathcal{D}^*=p_{SD}^*+p_{FD}^*+p_{DD}^*$) where the ratio value in equilibrium equals $\rho ^*/(1-\rho ^*)=p_\mathcal{H}^*/p_\mathcal{D}^*$.

The two-parameter bifurcation diagram $C$ versus $K$ for this reduced system Eq. (28) with $G$ given in Eq. (27c) is shown in Fig. 2.
